# Transforming growth factor-β1 requires NADPH oxidase 4 for angiogenesis *in vitro* and *in vivo*

**DOI:** 10.1111/jcmm.12263

**Published:** 2014-03-13

**Authors:** Hitesh M Peshavariya, Elsa C Chan, Guei Sheung Liu, Fan Jiang, Gregory J Dusting

**Affiliations:** a Centre for Eye Research Australia, Department of Ophthalmology, University of MelbourneEast Melbourne, Victoria, Australia; b O'Brien InstituteFitzroy, Victoria, Australia; c Key Laboratory of Cardiovascular Remodeling and Function Research, Qilu Hospital, Shandong UniversityJinan, Shandong Province, China

**Keywords:** NADPH oxidase, Nox4, angiogenesis, TGF

## Abstract

Angiogenesis, the formation of new blood vessels, is a key physiological event in organ development and tissue responses to hypoxia but is also involved in pathophysiologies such as tumour growth and retinopathies. Understanding the molecular mechanisms involved is important to design strategies for therapeutic intervention. One important regulator of angiogenesis is transforming growth factor-β1 (TGF-β1). In addition, reactive oxygen species (ROS) and the ROS-forming NADPH oxidase type 4 (Nox4) have been implicated as additional regulators such as during hypoxia. Here, we show that both processes are indeed mechanistically linked. TGF-β1-stimulated Nox4 expression and ROS formation in endothelial cells. In cells from Nox4-deficient mice, TGF-β1-induced cell proliferation, migration and tube formation were abolished. *In vivo*, TGF-β1 stimulated growth of blood vessels into sponges implanted subcutaneously, and this angiogenesis was markedly reduced in Nox4 knockout mice. Thus, endothelial cells are regulated by a TGF-β1 signalling pathway involving Nox4-derived ROS to promote angiogenesis. In order to abrogate pathological angiogenesis triggered by a multitude of factors, such as TGF-β1 and hypoxia, Nox4 may thus be an ideal therapeutic target.

## Introduction

Angiogenesis, the formation of new blood vessels from pre-existing capillaries, is fundamental to physiological processes such as embryonic development, wound healing, ischaemia-reperfusion and the menstruation cycle [1,2]. For example, ischaemia and the resultant hypoxia initiate an angiogenic response, which is important for tissue adaptation to hypoxia and ensuring sufficient oxygen delivery [3]. However, uncontrolled angiogenesis can also contribute to the pathogenesis of diseases such as diabetic retinopathy and tumour growth [1,2,4,5]. To allow therapeutic modulation, it is essential to identify the molecular mechanisms and signalling pathways involved in this angiogenesis. Recent evidence indicates that the angiogenic response to hypoxia depends on induction of growth factors such as VEGF and transforming growth factor β1 (TGF-β1). During hypoxia and angiogenesis, the up-regulation of both, TGF-β1 and VEGF appear to be mechanistically linked to a concomitant increase in the production of reactive oxygen species (ROS) [6–8], the source of which is unclear.

Transforming growth factor β1 is a multifunctional growth factor, which regulates many biological processes such as embryonic development, cell proliferation, migration, extracellular matrix production and differentiation of a variety of cell types. These diverse TGF-β1 responses are regulated *via* two trans-membrane serine/threonine kinase receptors, namely type I and type II. The cellular signalling pathway of TGF-β1 is initiated by its binding to its type II receptor and the subsequent recruitment of the type I receptor, also known as activin receptor-like kinase 5 (ALK5). The activated ALK5 induces phosphorylation of Smad2/3, which then binds to the regulatory subunit Smad4. This complex translocates to the nucleus, where it regulates the transcription of a specific set of genes involved in angiogenesis. In addition to ALK5, endothelial cells also express a type I receptor known as ALK1 that induces phosphorylation of Smas1/5 [9]. Transforming growth factor β1 has been implicated in the formation of new blood vessels. Indeed, it has been demonstrated that mice deficient in any of TGF-β1 [10], ALK5 [11], ALK1 [12] or Smad 2 [13] do not survive *in utero* for they suffer lethal defects in vasculogenesis and angiogenesis. These findings clearly indicate that TGF-β1 and its downstream signalling are important for regulation of angiogenesis.

Interestingly, TGF-β1 has been found to stimulate ROS production in a variety of cell types, including endothelial cells, *via* activation of NADPH oxidases [14–17], the only known enzymes whose sole function is to produce ROS [18,19]. There are seven isoforms of the NADPH oxidase catalytic subunit (Nox1 to Nox5, Duox1 and 2), among which Nox1, Nox2, Nox4 and Nox5 are expressed by human endothelial cells; the latter gene, however, is not present in rats or mice [20,21]. The different Nox isoforms can be distinguished by their sub-cellular localization, requirement for specific cytosolic subunits and activity regulation, the type of ROS produced and tissue-specific expression [18,20,22]. For example, the activation of Nox2 requires agonist-stimulated assembly of the cytosolic subunits p47phox, p67phox and Rac1 [8,23]. Nox5 does not require cytosolic subunits and, as a unique feature of the Nox subfamily, is the only one that is directly activated by calcium [24]. Nox4 is also unique in terms of its constitutive activity and that hydrogen peroxide (H_2_O_2_) rather than superoxide is its primary detectable product [25]. Recent studies indicate that during adaptation to hypoxia both *in vitro* and *in vivo* enhanced Nox4 expression is linked to angiogenesis involving different molecular pathways, such as eNOS [26] and HIF-1α [7]. Similarly, it is well-established that in several cell types including vascular smooth muscles cells [16] and fibroblasts [15], TGF-β1 induces H_2_O_2_ formation *via* Nox4. However, the functional link of this observation to angiogenesis has not been established. Here, we explore the role of Nox4 in TGF-β1-induced angiogenesis both *in vitro* and *in vivo* by using Nox4-deficient mice.

## Materials and methods

### Cell culture

Human umbilical vein endothelial cells (HUVEC), were purchased from Lonza, Victoria Australia, and human microvascular endothelial cells (HMECs) were kind gifts from Centre for Disease Control and Prevention, Atlanta, USA. All cell types were cultured in Endothelial Growth Medium-2 (EGM-2) Bullet Kit (hydrocortisone, gentamicin, amphotericin-B, VEGF, human epidermal growth factor, human fibroblast growth factor-basic (bFGF), R3 insulin like growth factor-1, ascorbic acid) with 15% foetal bovine serum (FBS; Lonza, Vic., Melbourne, Australia) in a 5% CO_2_ incubator at 37°C. Unless otherwise specified, cells were treated with TGF-β1 (10 ng/ml; Sigma-Aldrich, Castle Hill, NSW, Australia) for 6 hrs before cell harvest. When the effects of inhibitors were examined, cells were pre-treated with SB431542 (10 μM, ALK5 inhibitor; Sigma-Aldrich), for 1 hr before stimulation with TGF-β1 (10 ng/ml).

### Mouse heart endothelial cells isolation

Five mouse hearts were excised and collected in a tube containing cold DMEM with 2× Penicillin–Streptomycin antibiotics (Life Technologies, Mulgrave, Vic., Australia). Mouse hearts were pooled and washed three times with DMEM. Hearts were finely minced to ∽1 mm^3^ size parts with a scalpel and then mixed with 1 mg/ml type 1 collagenase (Sigma-Aldrich) as well as 1 mg/ml Dispase (Roche Diagnostics, Castle Hill, NSW, Australia) in DMEM. Hearts were digested for 1 hr at 37°C with agitation. Digested tissues were gently pipetted up and down for 10 times to break up clumps and then filtered through a 70-μm cell strainer (BD Biosciences, Sydney, NSW, Australia). The digested filtrate was centrifuged at 300 × *g* for 10 min., and the pellet was re-suspended in 2% EGM-2 media. The filtrate was incubated for 20 min. at 4°C with 10 μl of magnetic beads (Life Technologies) conjugated with anti-mouse CD31 antibody (BD Biosciences). Cells attached to the beads were collected by using a MPC magnet (Dynal Biotech, Life Technologies) and washed vigorously five times in 0.1% bovine serum albumin/PBS. Washed cells were collected and seeded in 60-mm tissue culture plates that had been pre-coated with fibronectin. The cells were grown in EGM-2 Bullet Kit with 15% FBS in a 5% CO_2_ incubator at 37°C. Cell growth was monitored and purity assessed by 1,1-dioctadecyl-3,3,3,3-tetramethylindocarbocyanine acetylated low-density lipoprotein (Dil-ac-LDL; Molecular Probe, Life Technologies) and expression of eNOS by real-time PCR.

### Adenovirus infection

We inhibited Nox4 gene expression by using adenoviral vectors expressing small interfering RNA targeting human Nox4 nucleotides 418–436 from the start codon (Adv-Nox4i) as described previously [27]. Adenovirus expressing green fluorescent protein (Adv-GFP) was used as a control. Cells were infected with 500 MOI (HUVECs) or 200 MOI (HMECs) of Adv-GFP or Adv Nox4 RNAi for 24 hrs in Opti-MEM media (Life Technologies) and allowed to recover in EGM-2 growth media for another 24 hrs. All experiments were performed 48 hrs after infection.

### Amplex red assay

Extracellular H_2_O_2_ levels were detected by using Amplex® Red assay kit (Molecular Probes, Life Technologies) according to manufacturer's instructions. Cells (10^5^ cells/well) were seeded in a 6-well plate. Serum-deprived cells were treated with and without TGF-β1 (10 ng/ml) for 6 and 24 hrs. Following treatments, trypsinized cells were suspended in Krebs-HEPES buffer (HBSS, in mM: NaCl 98.0, KCl 4.7, NaHCO_3_ 25.0, MgSO_4_ 1.2, 137 KH_2_PO_4_ 1.2, CaCl_2_ 2.5, d-glucose 11.1 and Hepes-Na 20.0) containing Amplex® Red reagent (10 mM) and horseradish peroxidase (0.1 U/ml). Fluorescence was then measured with excitation and emission at 550 and 590 nm, respectively, with a Polarstar microplate reader (BMG Labtech, Ortenberg, Germany) at 37°C. Fluorescence values were normalized to cell numbers determined by Alamar® Blue cell viability assays as according to manufacturer's instructions (Life Technologies).

### Dichlorodihydrofluorescein diacetate assay

Intracellular total ROS levels were detected by using 2′,7′-dichlorodihydrofluorescein diacetate (DCFH_2_-DA; Molecular Probes, Life Technologies) as described previously [28]. Cells (10^5^ cells/well) were seeded in a 6-well plate. Serum-deprived cells were treated with and without TGF-β1 (10 ng/ml) for 6 and 24 hrs. Following treatments, trypsinized cells were suspended in Krebs-HEPES buffer containing DCFH_2_-DA (10 μM). Fluorescence was then measured with excitation and emission at 490 and 530 nm, respectively, with a Polarstar microplate reader (BMG Labtech) at 37°C.

### Cell proliferation assay

Cells (10^4^ cells/well) were seeded in a 24-well plate. Serum-deprived cells were treated with and without TGF-β1 (10 ng/ml) for 24 hrs. Cell proliferation was then induced by replacing serum-free media with EGM-2 containing 1% FBS and TGF-β1 (10 ng/ml). After 48 hrs, cell numbers were analysed by using the Alamar® blue assay kit. Each well was incubated with Alamar® blue assay solution (Life Technologies, 1:10 dilution with EGM-2 media) for 1 hr at 37°C, 5% CO_2_. Fluorescence was measured with excitation and emission wavelengths of 480 and 520 nm, respectively by using a Polarstar microplate reader at 37°C.

### Wound healing assay

The wound healing assay is an *in vitro* model to explore effects on endothelial cell proliferation and migration during closure of a cellular monolayer wound. HUVECs (10^5^ cells/well) or HMECs (1.5 × 10^5^) were seeded in 12-well plates; murine heart endothelial cells (MHECs), in 96-well plates. After 24 hrs, two perpendicular wounds were created by using 1 ml and 200 μl pipette tips for HUVECs/HMECs and MHECs, respectively. Cells were washed three times with PBS and treated with EGM-2 media containing 2% FBS in the absence and presence of TGF-β1 (10 ng/ml) for 16 hrs at 37°C, 5% CO_2_. Images were captured under 10× magnification when the wound was made (time zero) and at 16 hrs. Three different areas of the wound were measured with Image J software. The investigator was blinded to the treatment groups. Values were then expressed as the percentage wound recover at time zero and at 16 hrs.

### Tube formation assay

Serum-deprived cells (1.5 × 10^4^ cells/well) were seeded on growth factor-reduced Matrigel (50 μl) in 96-well plate. Cells were treated with or without TGF-β1 (10 ng/ml) in the presence of EGM-2 media containing 2% FBS at 37°C, 5% CO_2_. After 8 hrs, images were taken under 4× and 10× magnifications by using an Olympus inverted light/fluorescent microscope (model no. IX81, Albertsturd, Denmark). Complete loop formation was counted from the entire well and normalized to controls. The investigator was blinded to the treatment groups.

### Gene expression analysis

Endothelial cells (10^5^ cells/well) were seeded in 6-well plates and serum deprived overnight before experiments. Total RNA from treated cells was extracted with the TRI reagent according to manufacturer's instructions (Ambion, Austin, TX, USA) and reverse-transcribed to cDNA by using TaqMan high performance reverse transcription reagents (Applied Biosystems, Life Technologies) at 25°C for 10 min., 37°C for 2 hrs followed by 85°C for 5 s in a Thermal cycler (BioRad-DNA Engine; Bio-Rad, Gladesville, NSW, Australia). The real-time PCR reactions were performed in a 7300 system (Applied Biosystems, Life Technologies) by using TaqMan Universal PCR master mix and pre-designed gene specific probes and primer sets for Nox2 (Hs00166163_m1), Nox4 (Hs01558199_m1 and Mm00479246_m1) and NOS3 (Hs00167166_m1and Mm00435204_m1). Data were normalized to GAPDH (human 4326317E and mouse 4352339E) and expressed as fold changes over that in control treatment group.

### Western blot analysis

Cells (10^5^cells/well) were cultured in 6-well plates, and protein was extracted as previously described [29]. Primary rabbit polyclonal anti Nox4 (1:1000, kindly provided by Prof. Ajay M. Shah), pSmad2 (1;1000; Calbiochem, Merck Millipore, Billerica, MA, USA), total Smad (1: 1000; Abcam, Waterloo, NSW, Australia), and mouse monoclonal β-actin (1:4000; Sigma-Aldrich) antibodies were used. Proteins were detected with enhanced chemiluminescence detection kit (GE Healthcare, Sydney, NSW, Australia) with horseradish peroxidase conjugated to appropriate secondary antibodies (Bio-Rad). The GeneGenius Imaging System from Syngene was used to capture the images.

### *In vivo* angiogenesis

Animal study has been conducted in accordance with St. Vincent's Hospital Animal Ethics Committee guidelines (Melbourne, Victoria, Australia) and the Australian National Health and Medical Research Council guidelines for the care and health of animals. The subcutaneous sponge model was used to determine the effects of TGF-β1 (10 ng/ml) on angiogenesis *in vivo*. UV-sterilized polyvinyl alcohol (PVA) sponge discs (8 mm diameter × 2 mm thickness from PVA Unlimited, USA) were soaked in either saline (120 μl/sponge) or TGF-β1 solution (10 ng/ml; 120 μl/sponge) and implanted under the dorsal skin of 8-12 week old male C57BL6 wild-type mice, Nox4 knockout mice (kindly provided by Prof. Harald HHW Schmidt), [30], and Nox2 knockout mice (The Jackson Laboratory, Bar Harbor, ME, USA). After 14 days, sponges were harvested and cleaned of connective tissues. Sponges were fixed in 4% paraformaldehyde overnight, processed and sectioned (4 μm) for immunohistochemical analysis. For haemoglobin assays, sponges were incubated with 500 μl of red blood cells lysis buffer (in mM; NH_4_Cl 200, NaHCO_3_ 20, ethylenediaminetetraacetic acid 1) for 1 hr at 37°C. The supernatant was collected by centrifugation at 5000 × g for 10 min. The concentration of haemoglobin in the supernatant was determined at an absorbance of 550 nm and compared with a standard curve of purified bovine haemoglobin (Sigma-Aldrich) with a haemoglobin assay kit (Drabkin's reagent; Sigma-Aldrich).

### Identification of capillary in fixed sponge sections with CD31 antibody

Sponge sections were digested by 0.1% Proteinase K (pH 7.8) at 37°C for 3 min. Endogenous peroxidase activity was quenched by 3% H_2_O_2_. The sections were blocked with total protein block solution for 20 min. (Dako, North Sydney, NSW, Australia ) and then incubated with rat anti-mouse CD31 (1:150, MEC13.3, BD Biosciences Pharmingen) for 1 hr. Slides were washed with PBS and incubated with biotinylated rabbit anti-rat IgG (1:200; Vector Laboratories, East Brisbane, QLD, Australia) for 30 min., and developed with an ABC kit (Vector Laboratories) and DAB chromogen (Dako). Slides were counterstained with haematoxylin. Normal rat IgG was used instead of primary antibody as negative control. CD31 positive counts were assessed with the computer-assisted stereological toolbox system (Olympus). By using an eyepiece with a grid, CD31 positive vascular density (% of area) were estimated by the number of points that fell randomly on CD31 positive staining divided by the total number of points counted.

### Data and statistics

Data are expressed as mean ± SEM. The mean data were analysed with Student's *t*-test or one-way anova followed by *post hoc* Tukey analysis. A value of *P* < 0.05 was regarded as statistically significant.

## Results

### TGF-β1 up-regulates Nox4 expression in vascular endothelial cells

**Figure 1:**
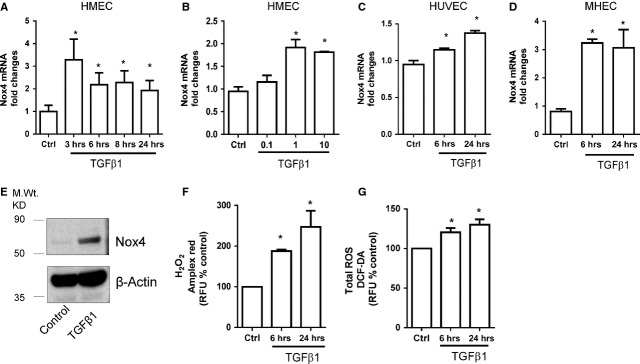
Transforming growth factor-β1 (TGF-β1) increases Nox4 mRNA and protein levels as well as reactive oxygen species (ROS) generation in endothelial cells. TGF-β_1_ induced Nox4 mRNA levels in a time- (A; 10 ng/ml; 3–24 hrs) and concentration-dependent (B; 0.01–10 ng/ml) manner in HMECs. (C) TGF-β1 (10 ng/ml) also induced Nox4 gene expression at 6 and 24 hrs in human umbilical vein endothelial cells (HUVECs) and (D) murine heart endothelial cells (MHEC). (E) TGF-β1 (10 ng/ml) enhances Nox4 protein levels compared with control in HMECs as shown in a representative Western blot by using a specific Nox4 antibody. (F) H_2_O_2_ and (G) Total ROS generation as detected by using Amplex red and DCFH_2_-DA respectively by HMECs were increased following 6 and 24 hrs stimulation of TGF-β (10 ng/ml). All data are mean ± SEM from three to five experiments, *P < 0.05 from control (Ctrl).

First, we examined the effects of different growth factors involved in angiogenesis on endothelial expression of Nox2 and Nox4. Human microvascular endothelial cells were treated with VEGF, bFGF, Platelet-Derived Growth Factor BB (PDGF-BB) and TGF-β1 (all at 10 ng/ml) for 3–24 hrs. Only TGF-β1 consistently up-regulated the expression of Nox4, whereas Nox2 expression was not changed by any of the growth factors (Figure S1 and Fig.1A). Nox1 and Nox5 were below the detection limit. PCR conditions were validated by using the colorectal cancer cell line caco-2 and the prostate cancer cell line LNCaP as a positive control for Nox1 and 5, respectively (data not shown). Next, we analysed the concentration-dependent stimulatory effect of TGF-β1 on Nox4 expression by treating the cells with different concentrations of TGF-β1 for 6 hrs. As shown in Figure1B, the TGF-β1 has similar effect on Nox4 expression at 1 and 10 ng/ml whereas lowest concentration of TGF-β1 (0.1 ng/ml) did not affect Nox4 expression. To confirm that the TGF-β1-induced effects on Nox4 expression were not specific to HMECs, we treated primary culture of HUVEC and MHEC derived with TGF-β1 (10 ng/ml) for 6 and 24 hrs. Indeed, TGF-β1 induced Nox4 expression also in both HUVEC and MHEC (Fig.1C and D). In accordance, TGF-β1 (10 ng/ml) also increased Nox4 protein levels in HMECs (Fig.1E). Finally, we showed that TGF-β1 treatment elevated H_2_O_2_ and total ROS production (Fig.1F and G) in HMECs.

### TGF-β1 stimulates Nox4 expression *via* the Smad2 pathway

**Figure 2:**
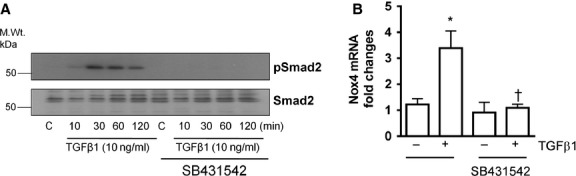
Transforming growth factor-β1 (TGF-β1) increases Nox4 gene expression through Smad-dependent pathway in HMECs. (A) Representative Western blot showing the effects of TGF-β1 (10 ng/ml) on Smad2 phosphorylation. Blocking the effect of Activine Link kinase (ALK) with SB431452 (10 μM) inhibits Smad2 phosphorylation following TGF-β1 (10 ng/ml) stimulation. (B) The stimulatory effect of TGF-β1 (10 ng/ml) on Nox4 gene expression is inhibited by SB431452 (10 μM). All data are mean ± SEM from three to four experiments, *P < 0.05 from control without treatment; †*P* < 0.05 from cells treated with TGF-β1 (10 ng/ml).

One of the earliest events of TGF-β1 signal activation is phosphorylation of Smad2/3 and its translocation to nucleus to activate gene expression. To understand the signalling pathways involved in TGF-β1-induced up-regulation of Nox4 in endothelial cells, we therefore explored the role of TGF-β1 receptor signalling Smad proteins. As expected, phosphorylation of Smad2 was increased by TGF-β1 from 30 min. and persisted up to 120 min., whereas total Smad2 protein levels were unchanged. To clarify whether TGF-β1-induced effects were dependent on TGF-β1 receptors I ALK5 activity, we co-treated the cells with TGF-β1 and the inhibitor of its receptor ALK5, SB431542 (10 μM). Consistent with previous studies, TGF-β1-induced Smad2 phosphorylation (Fig.2A) and Nox4 up-regulation (Fig.2B) were blocked by the ALK5 inhibitor.

### Nox4 is required for TGF-β1-induced H_2_O_2_ generation

**Figure 3:**
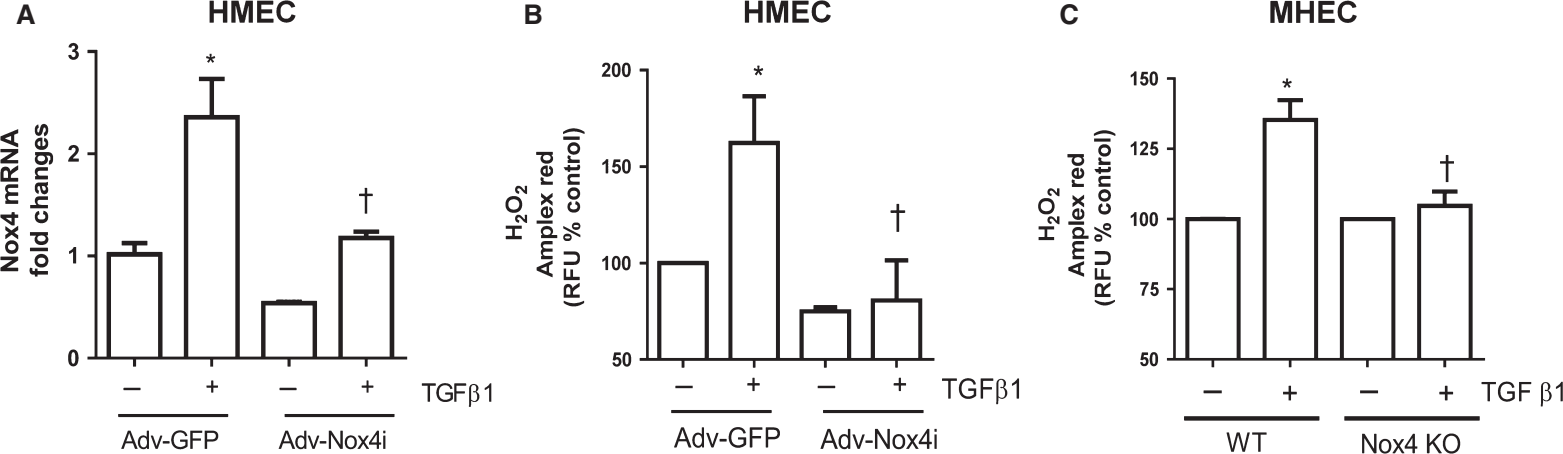
Transforming growth factor-β1 (TGF-β1) regulates Nox4-derived H_2_O_2_ in endothelial cells. Treatment of HMECs with Adv-Nox4i suppressed the stimulatory effects of TGF-β1 (10 ng/ml; A) on Nox4 gene expression and (B) H_2_O_2_ generation. (C) TGF-β1 (10 ng/ml)-induced H_2_O_2_ generation was abolished in MHEC derived from Nox4 knockout (Nox4 KO) mice. All data are mean ± SEM from three to six experiments, *P < 0.05 from control without treatment; †*P* < 0.05 from Adv-GFP or WT-treated cells in the presence of TGF-β1 (10 ng/ml).

To illustrate the functional importance of TGF-β1-induced Nox4 up-regulation, we used an adenovirus carrying RNA interference targeting human Nox4 (Adv-Nox4i) to down-regulate Nox4 in HMECs. As expected, Adv-Nox4i markedly reduced TGF-β1-stimulated Nox4 mRNA expression in HMECs (Fig.3A). Importantly, we also showed that Adv-Nox4i suppressed H_2_O_2_ production in the presence of TGF-β1 stimulation (Fig.3B). To further confirm that TGF-β1-mediated H_2_O_2_ generation was dependent on Nox4 gene expression, we isolated heart endothelial cells (MHEC) from Nox4 knockout mice (Nox4 KO) and their wild-type littermates (WT). Murine heart endothelial cells and HUVECs were found to share some similar characteristics as demonstrated in Figure S2. Both cell types exhibited cobblestone morphology, uptake of Dil-Ac-LDL and formed tubes when they were suspended in growth factor containing Matrigel. The gene expression of eNOS was also found to be similar in both cell types (Figure S2), confirming the homogeneity of the isolated MHECs. As shown in Figure3C, MHEC from WT mice also responded to TGF-β1 with an increased H_2_O_2_ production, and this response reduced in Nox4 KO mouse-derived MHEC. This confirms that Nox4 is required for TGF-β1-induced H_2_O_2_ formation in endothelial cells.

### Nox4 is involved in TGF-β1-induced endothelial cell proliferation

**Figure 4:**
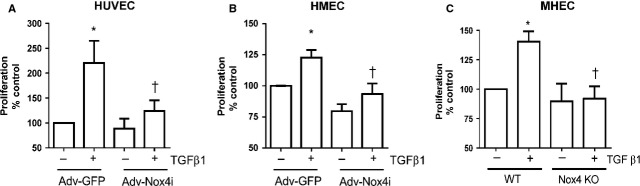
Nox4 is required for transforming growth factor-β1 (TGF-β1)-stimulated endothelial cell proliferation. TGF-β1 (10 ng/ml) increased cell proliferation of (A) HUVEC (B) HMECs. Adv-Nox4i infection suppressed TGF-β1-induced cell proliferation of both cell types. (C) Similarly, TGF-β1 (10 ng/ml) induced cell proliferation wild-type (WT)-derived MHEC, and this effect was reduced in MHEC derived from Nox4 knockout (Nox4 KO) mice. All data are expressed as mean ± SEM from three to four experiments. Cell proliferation is expressed as a percentage of Adv-GFP control or WT without TGF-β1 treatment. *P < 0.05 from Adv-GFP without TGF-β1 treatment; †*P* < 0.05 from Adv-GFP or WT treatment in the presence of TGF-β1.

Previously, we and others have showed that Nox4 enhanced endothelial cell proliferation [27,31]. We therefore attempted to clarify whether Nox4 has a role in TGF-β1-mediated endothelial cell proliferation. As expected, TGF-β1 increased cell proliferation in HUVEC and HMEC. Importantly, this effect was prevented by knocking down Nox4 by using Adv-Nox4i in both HUVECs (Fig.4A) and HMECs (Fig.4B). To confirm that TGF-β1-mediated proliferation responses were dependent on Nox4, we used MHEC from Nox4 KO and their wild-type littermates (WT). There was no difference in the degree of cell proliferation between WT and Nox4 KO at the basal levels. However, in consistence with the inhibitory effect of Ad-Nox4i on TGF-β1-induced endothelial cell proliferation, the stimulatory effect of TGF-β1 on cell proliferation was abolished in Nox4 KO cells (Fig.4C). These findings clearly show that Nox4 is required for TGF-β1-induced endothelial cell proliferation.

### Nox4 is required for TGF-β1-induced angiogenic responses *in vitro*

**Figure 5:**
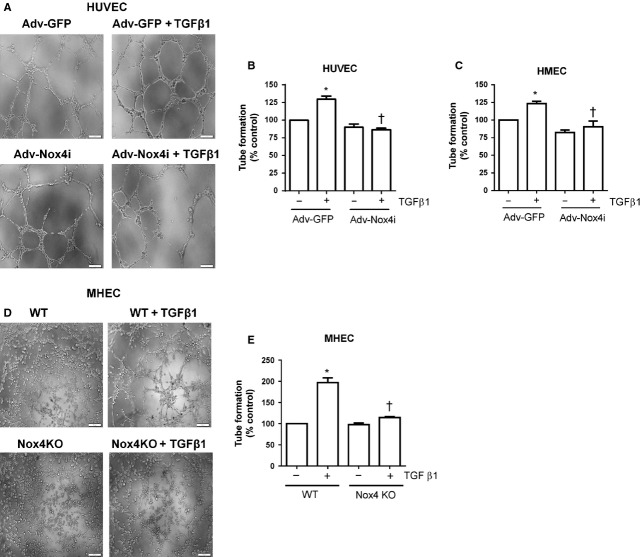
Nox4 is required for transforming growth factor-β1 (TGF-β1)-induced endothelial cell capillary formation. Adv-Nox4i abrogated the stimulatory effect of TGF-β1 (10 ng/ml) on capillary formation of (A and B) in HUVECs and (C) HMECs in growth factor reduced Matrigel. Similarly, TGF-β1 (10 ng/ml) induced capillary formation within 8 hrs of application in MHEC derived from wild-type (WT), and this response was abrogated in Nox4 knockout mouse cells (Nox4 KO; D and E). Representative high magnification images of tube formation assay performed on (A) HUVECs and (D) MHEC (scale bar represents 100 μm). Quantitative measures (mean ± SEM from *n* = 6) of capillary formation are expressed as a percentage of cells infected with Adv-GFP controls or WT cells in the absence of TGF-β1. *P < 0.05 from Adv-GFP or WT without TGF-β1 treatment; †*P* < 0.05 from Adv-GFP or WT treatment in the presence of TGF-β1.

In other studies TGF-β1 has been suggested to promote angiogenesis [32,33]. To explore the functional significance of TGF-β1 in regulation of Nox4, we therefore examined the effect of TGF-β1 on angiogenic responses of endothelial cells *in vitro* and then *in vivo*. Formation of capillary-like structures was assessed by plating HUVEC and HMECs on solidified growth factor-reduced Matrigel in the absence of serum. Within 8 hrs, TGF-β1-treated cells formed capillary-like structures more efficiently than untreated cells as shown in representative pictures of HUVEC in Figure5A. Transforming growth factor β1-enhanced capillary-like structure formation was indeed prevented by Adv-Nox4i treatment in HUVEC and HMECs (Fig.5B and C). To confirm that Nox4 is required for the TGF-β1-induced angiogenesis, we also studied MHECs. Similar as in human endothelial cells, TGF-β1 enhanced capillary-like structures in WT mouse-derived MHEC suspended in Matrigel, and this response was abolished in Nox4 KO mouse-derived MHEC (Fig.5D and E). These findings suggest that TGF-β1-induced Nox4 plays a role in the formation of endothelial capillary-like structures by endothelial cells, at least *in vitro*.

### Nox4 is important for TGF-β1-promoted endothelial wound healing responses *in vitro*

**Figure 6:**
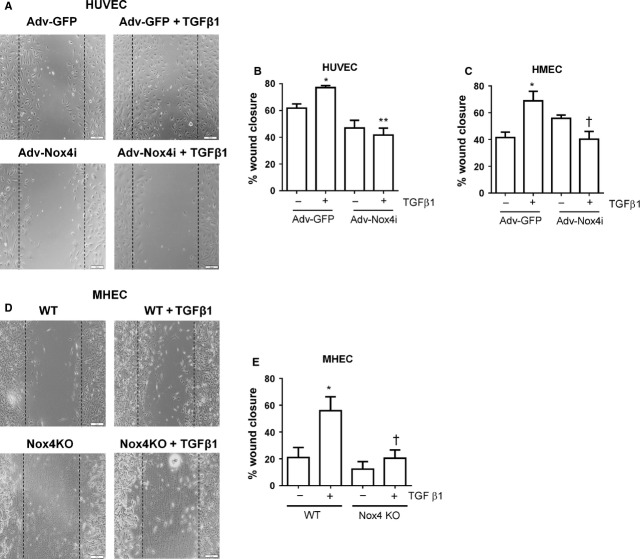
Nox4 is required for transforming growth factor-β1 (TGF-β1)-induced wound healing. Adv-Nox4i blunted the stimulatory effect of TGF-β1 (10 ng/ml) on wound healing response of (A and B) HUVECs and (C) HMECs. Similarly, TGF-β1 (10 ng/ml) induced wound healing response in wild-type mouse cells (WT), and this response was blunted in Nox4 KO MHEC (C and D). Representative high magnification images of a single wound-scratched healing assay performed on (A) HUVECs and (D) MHEC (scale bar represents 100 μm). Quantitative measures (mean ± SEM from *n* = 3–6) of wound healing responses are expressed as a percentage of cells infected with Adv-GFP controls or WT without TGF-β1 treatment. *P < 0.05 from Adv-GFP without TGF-β1 treatment; †*P* < 0.05 from Adv-GFP or WT treatment in the presence of TGF-β1.

Disruption of the intact layer of endothelial cells by using a scratch assay model causes migration and proliferation of adjacent cells to fill in the wounded areas. As expected, TGF-β1 accelerated the wound closure in Adv-GFP infected HUVEC and HMECs (Fig.6A–C). Importantly, TGF-β1-induced wound recovery was abolished by Adv-Nox4i (Fig.6A–C). Similarly, TGF-β1 increased the wound closure in wild-type MHEC when compared with untreated endothelial cells (Fig.6D and E), and this wound recovery response was decreased in Nox4 KO-derived mouse MHEC (Fig.6D and E). These data suggest that TGF-β1 promoted migration and wound closure responses of endothelial cells depend on Nox4.

### Nox4 mediates TGF-β1- induced angiogenesis *in vivo*

**Figure 7:**
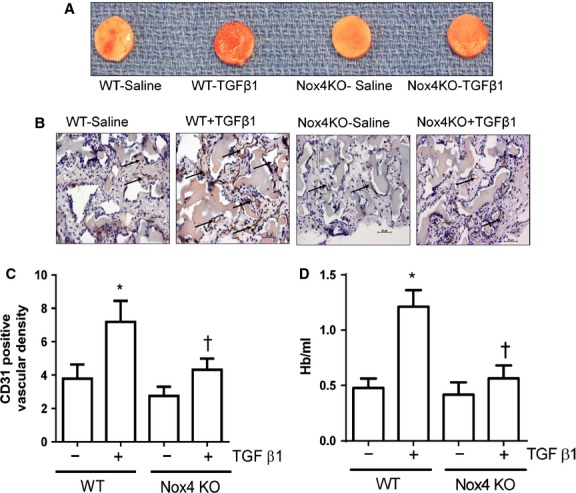
Nox4 is required for transforming growth factor-β1 (TGF-β1)-induced angiogenesis *in vivo*. Representative picture of polyvinyl alcohol sponges excised from wild-type (WT) and Nox4 knockout (Nox4 KO) mice after 14 days (A). Images of fixed sponge sections show positive brown stains (indicated by arrow) to the endothelial marker CD-31 (B). TGF-β1-induced increases in CD-31 counts in wild-type (WT) were almost abolished in Nox4 KO (C). Haemoglobin content of sponges as an index of angiogenesis and was expressed as Hb/ml (mean ± SEM from *n* = 8). *P < 0.05 from wild-type with saline treatment; †*P* < 0.05 from wild-type with TGF-β1 treatment.

So far we have shown that Nox4 is essential for TGF-β1-mediated angiogenic responses in endothelial cells, *in vitro*. We therefore explored the role of Nox4 in TGF-β1-induced angiogenic responses with an *in vivo* sponge model. We instilled sponges with saline or TGF-β1 (10 ng/ml) and implanted them subcutaneously in the dorsal skin of WT and Nox4 KO mice for 14 days to allow vessels to grow into the sponges. The degree of vessel growth in the sponges was then evaluated by immunohistochemical detection of CD31 positive vessels in fixed sponge section and by quantification of haemoglobin content in sponges. As demonstrated in Figure7A, TGF-β1-treated sponges harvested from WT mice appeared to have retained more blood when compared with the saline treatment or saline- and TGF-β1-instilled sponges harvested from Nox4 KO mice. Similarly, TGF-β1 treatment significantly enhanced the degree of CD31-identified vessels in sponges harvested from WT mice, and this response was abolished in Nox4 KO mice (Fig.7B and C). Moreover, TGF-β1-treated sponges obtained from WT mice showed higher haemoglobin content (Fig.7D) compared with saline-treated sponges, suggesting that TGF-β1-induced vessels were perfused with blood. Most importantly, the augmenting effects of TGF-β1 on haemoglobin content were almost completely abolished in Nox4 KO mice (Fig.7C and D) but not in Nox2 KO mice (Figure S3). Interestingly, there was no difference in the degree of blood vessel formation and haemoglobin content in the sponges between Nox4 KO and WT mice under basal conditions, suggesting that Nox4 regulates angiogenesis upon stimulation with TGF-β1. Collectively, our findings suggest that Nox4 is required for TGF-β1-induced angiogenesis not only *in vitro* but also *in vivo*.

## Discussion

Here, we show that TGF-β1 up-regulates Nox4 expression in endothelial cells and promotes angiogenesis. Nox4-derived ROS signalling is involved in many salient features of angiogenic processes such as endothelial cell proliferation, migration and tube formation. These responses were absent in Nox4-deficient endothelial cells. Importantly, deletion of Nox4 *in vivo* markedly reduced angiogenesis in response to TGF-β1 in subcutaneous sponges.

Transforming growth factor β1-mediated activation of Smad2/3 has been implicated in the induction of Nox4 and ROS formation in several cell types. Previously, it has been shown that TGF-β1 consistently up-regulates Nox4 expression *via* Smad 2/3-dependent pathways in fibroblasts, human pulmonary artery smooth muscle cells and breast cancer cells [15,34,35]. To define how Nox4 expression is regulated in endothelial cells, we tested the effects of the pharmacologic ALK5 inhibitor SB431542, which blocked TGF-β1-induced phosphorylation of Smad2 and Nox4 expression in endothelial cells, consistent with previous findings [15,34,35]. These findings support a role of TGF-β1 signalling *via* ALK5 and Smad2/3 for Nox4 expression in endothelial cells.

Adaptation to ischaemia and angiogenesis induced by growth factors may require NADPH oxidase-derived ROS signalling. Previously, it was shown that Nox2 is involved in angiogenesis induced in hindlimbs by ischaemia or by VEGF [36,37] and hypoxia/VEGF-driven angiogenesis in retina [38], although it is important to note that angiogenesis was not abolished in Nox2-deficient mice. Recently, two independent studies demonstrated that Nox4-derived ROS signalling is also important for ischaemia-induced angiogenesis *in vivo* [26,39]. The role of Nox1 in angiogenesis is less clear, and we could not detect Nox1 mRNA in human endothelial cells by using commercially available primers and probes. Moreover, in murine heart endothelial cells, the expression of Nox1 did not change after TGF-β1 treatment (data not shown). In contrast, Schroder *et al*. showed that in Nox1 knockout mice ischaemia-induced angiogenesis was enhanced [39] and tumour-induced angiogenesis reduced [40]. These findings suggest that each Nox isoform contributes to angiogenesis *via* different downstream signalling pathways. Indeed, our data demonstrated that TGF-β1, but not other growth factors (VEGF, bFGF and PDGF) promoted Nox4 expression in endothelial cells. In addition, the TGF-β1-induced angiogenic response was unaffected in Nox2-deficient mice (Figure S3), but completely abolished in Nox4 KO mice. Thus, previous studies and our data imply that each Nox isoform is differentially regulated by different signalling molecules. In our hands, Nox4, but not Nox2, plays a pivotal role in the TGF-β1-driven formation of new blood vessels.

The mechanisms of TGF-β1-induced Nox4 expression and pro-angiogenic response in endothelial cells warrant further investigation. Recent reports suggest that several intracellular signalling pathways are regulated by Nox4-derived ROS through reversible inactivation of phosphatases (PTP1B) [27], activation of transcription factors (HIF-1α and Nrf2) [7,39,41] and up-regulation of gene expression (eNOS) [26]. These target proteins have been implicated in angiogenesis induced by either hypoxia or growth factors. In our study, basal expression of eNOS did not differ in either endothelial cells or subcutaneous sponges obtained from wild-type and Nox4 KO mice. Interestingly, the marked stimulatory effect of TGF-β1 on eNOS gene expression was abolished in sponges from Nox4 KO mice (Figure S4A) but was unaffected in endothelial cells isolated from Nox4 KO mice (Figure S4B), suggesting differences in regulation of TGF-β1-induced Nox4 gene up-regulation on eNOS expression between *in vitro* and *in vivo* conditions. Apart from eNOS, Nox4 has been found to regulate HIF-1α activity in the myocardium to promote compensatory angiogenesis following pressure overload in mice [7]. The role of HIF-1α in angiogenesis is well-established, and HIF-1α deficiency has been found to be lethal to embryo development because of vascular regression [42]. Furthermore, a recent study revealed that mice lacking endothelial HIF-1α died prematurely in a ventricular pressure overload model. In this model, TGF-β1 stimulated fibrosis and decreased myocardial angiogenesis *via* a Smad-independent pathway [43]. Thus, it is plausible that adaptation to hypoxia enhances TGF-β1-induced Nox4 *via* a Smad-dependent pathway, which may extend the stabilization of HIF-1α and enhances angiogenesis. Such a mechanism requires confirmation in our model.

Transforming growth factor β1-induced Nox4 has impacts on cell fate in a context-dependent manner, as ROS may contribute to cell survival, proliferation, hypertrophy or differentiation depending on the cell type and/or the concentration of ROS. Accordingly, it has been shown that TGF-β1 *via* Nox4 signalling induces apoptosis in epithelial cells [14] and hepatocytes [44] whereas it induces proliferation of fibroblasts [15]. Similarly, TGF-β1-induced Nox4 expression contributes to proliferation of human pulmonary artery smooth muscle cells and treatment with Nox4 siRNA decreases their proliferation [34,45]. Previously, only a few studies have shown an interaction between TGF-β1 and Nox4 in endothelial cells. For instance, acute treatment with TGF-β1 enhanced Nox4-derived ROS signalling *via* a Smad-independent mechanism, which contributes to cytoskeleton rearrangement in human endothelial cells [17]. In pulmonary and lung microvascular endothelial cells, chronic treatment with TGF-β1 also induces Nox4 transcripts, but the downstream consequences were not studied [34]. Our study not only demonstrates that in human and mouse endothelial cells chronic treatment with TGF-β1-induces Nox4 expression, but for the first time provides clear evidence that a TGF-β1-Nox4 pathway is essential to promote proliferation, migration and angiogenesis *in vitro* and *in vivo*.

Transforming growth factor β1 is a well-established regulator of endothelial cell proliferation, differentiation, vascular network formation and maintenance of vessel wall integrity [9]. Several studies have indicated that adverse effects of TGF-β1 signalling pathways include participation in pathology of fibrosis [14,15,46], neointima formation [47], as well as cancer progression and metastasis [35,48]. Given that TGF-β1 signalling has deleterious effects on many cell types, TGF-β1-induced Nox4 activation is not a suitable target to induce therapeutic angiogenesis. In contrast, inhibition of TGF-β1-induced Nox4 signalling may be useful to reduce pathological angiogenesis. Furthermore, direct interference with TGF-β1 signalling may lead to vascular disorders such as haemorrhagic telangiectasia [9,49]. Therefore, inhibition of Nox4 might be a better target to block the deleterious effects of TGF-β1 under pathological conditions. Indeed, recently it has been shown that GKT137831, an inhibitor of Nox1 and Nox4, attenuated hypoxia-induced TGF-β1 expression and proliferation of human pulmonary artery endothelial and smooth muscle cells *in vitro*, as well as attenuated pulmonary artery wall thickening and vascular remodelling *in vivo*, features which are all characteristic of idiopathic pulmonary hypertension [6].

**Figure 8:**
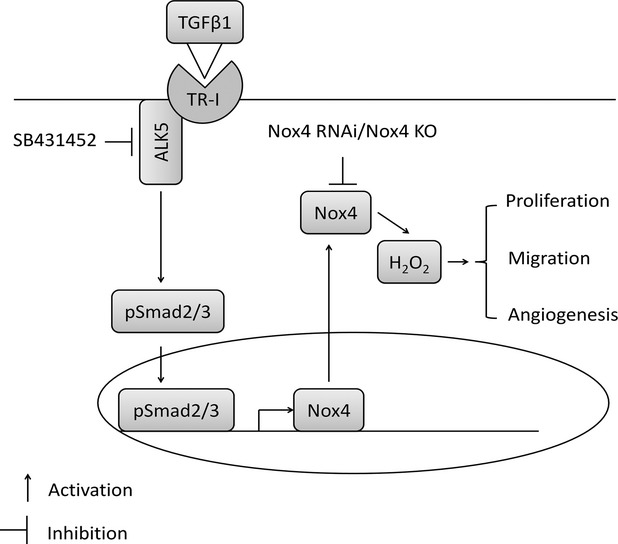
A diagram showing the potential mechanism of transforming growth factor-β1-induced Smad2/3 signalling pathway which may be involved in Nox4 up-regulation and angiogenesis.

In summary, we have shown that TGF-β1 stimulated Nox4 expression in endothelial cells, and demonstrated its significance for angiogenesis (Fig.8). Adaptation to hypoxia-induced secretion of different growth factors such as TGF-β1 and VEGF to increase neovascularization of ischaemic tissues is a hallmark of pathological angiogenesis in diabetic retinopathy and cancer. If several of these pro-angiogenic signalling pathways merge into Nox4, in such conditions of pathological angiogenesis Nox4 inhibition may be a superior therapeutic target than blocking TGF-β1 or VEGF individually.

## Supporting Information

**Figure S1** Effect of VEGF, bFGF and PDGF on Nox4 and Nox2 mRNA in endothelial cells.

**Figure S2** Characterization of primary mouse heart endothelial cells (MHEC).

**Figure S3** Nox2 is not required for TGF-β1-induced angiogenesis *in vivo*.

**Figure S4** eNOS mRNA expression was higher in sponges treated with TGF-β in wild-type mice (WT), and this effect was abolished in Nox4 KO mice (A).
