# Supplementary figures and images for: Transforming growth factor-β1 requires NADPH oxidase 4 for angiogenesis in vitro and in vivo
Source: J Cell Mol Med. 2014 Mar 13;18(6):1172–83. doi: 10.1111/jcmm.12263 (PMC4508156; doi:10.1111/jcmm.12263)

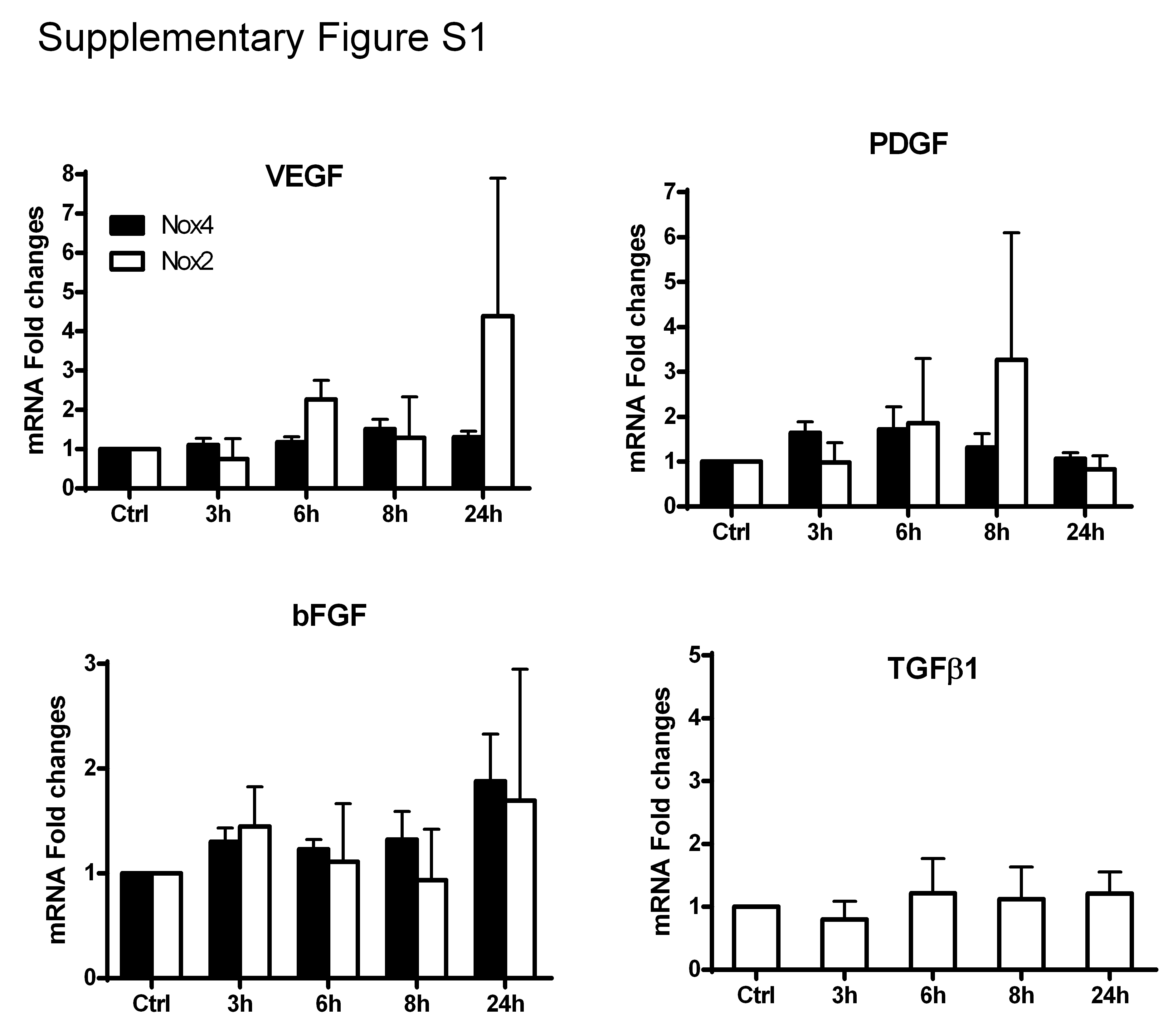

Supplement: Supplementary file 1 [file jcmm0018-1172-sd1.tif]

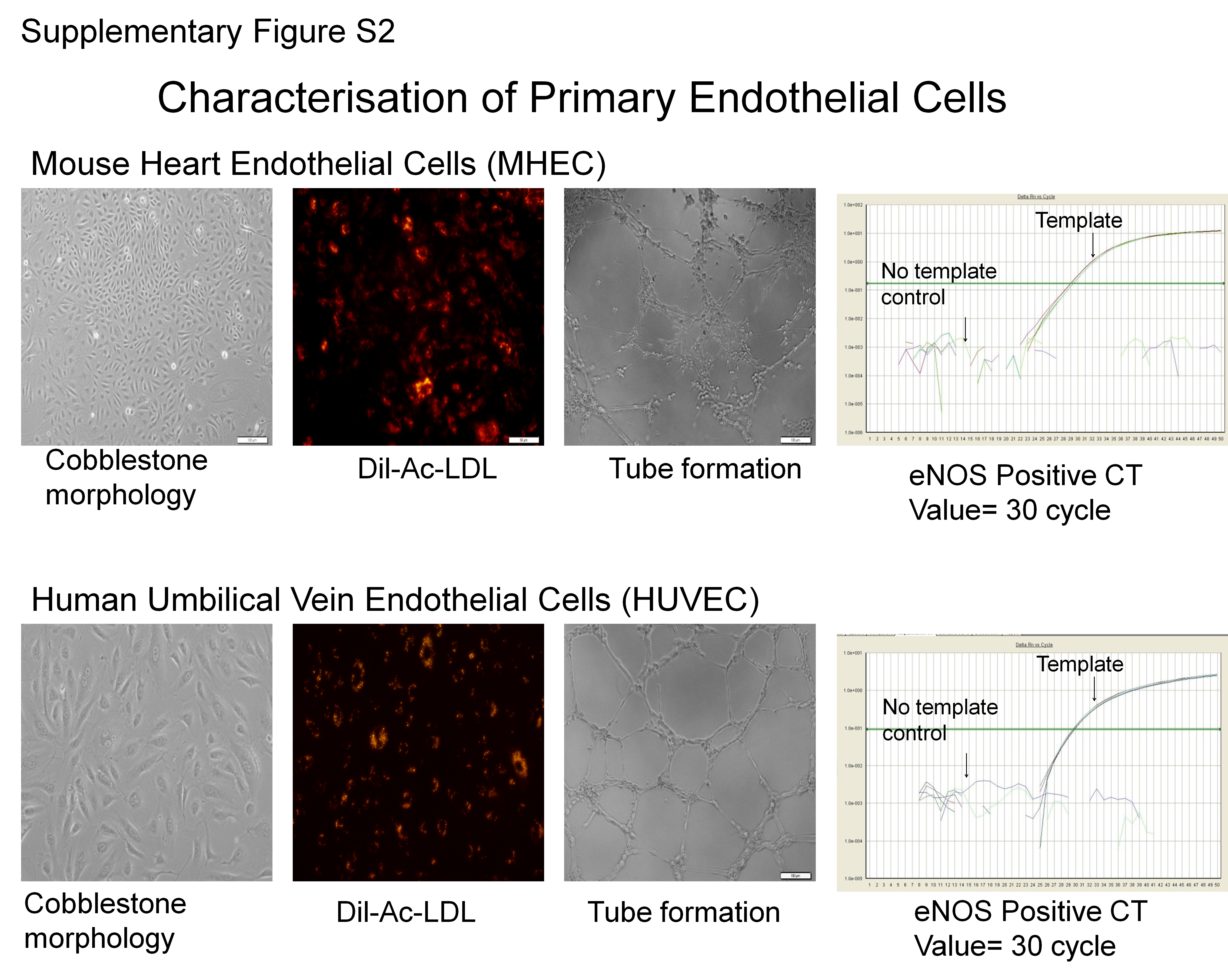

Supplement: Supplementary file 2 [file jcmm0018-1172-sd2.tif]

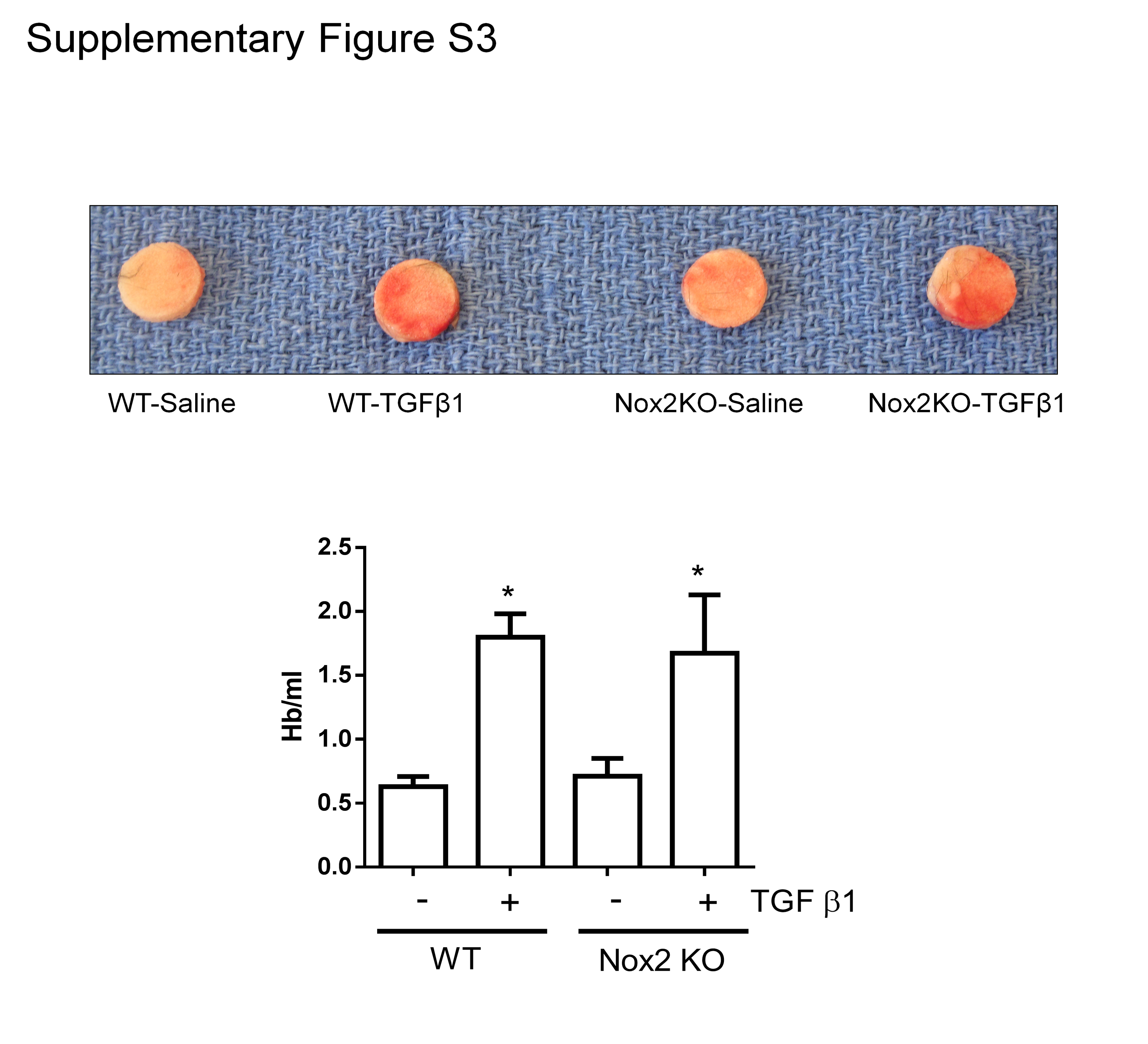

Supplement: Supplementary file 3 [file jcmm0018-1172-sd3.tif]

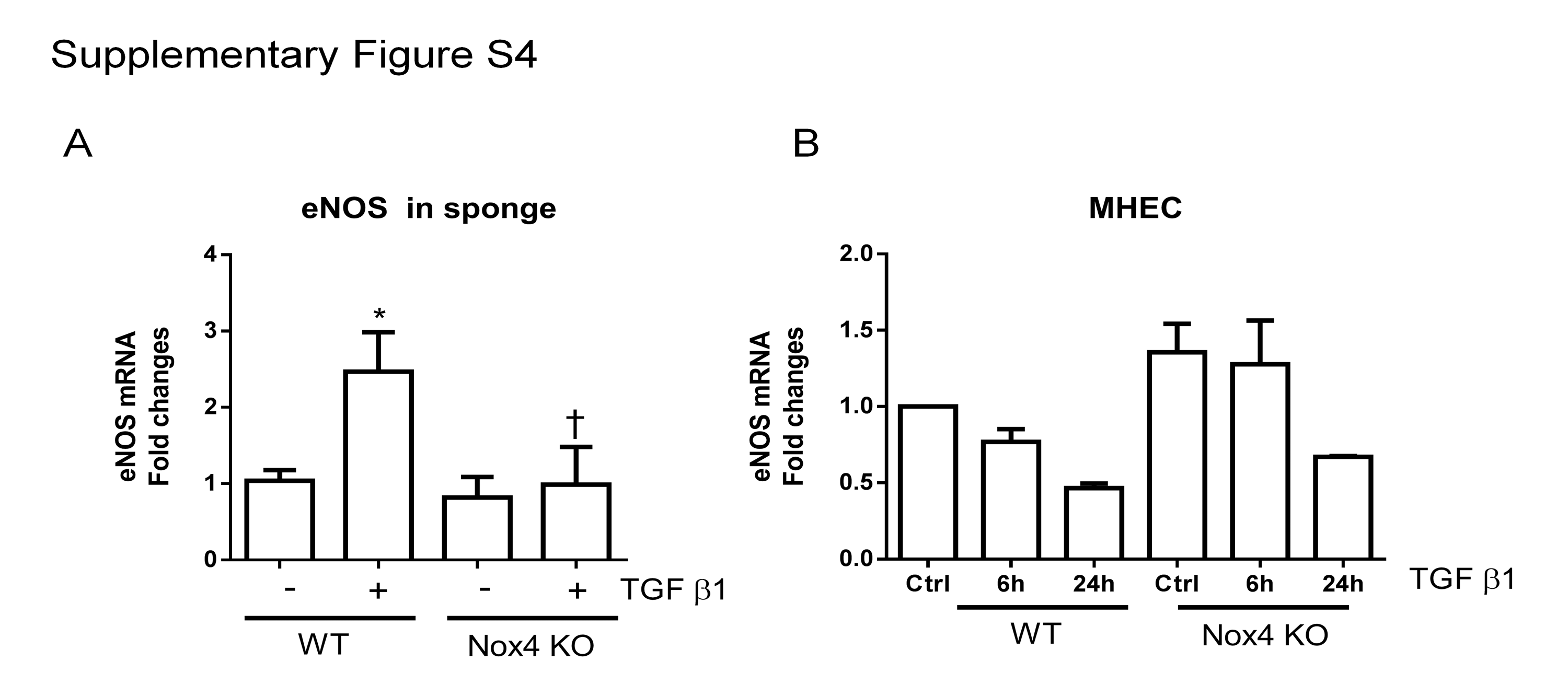

Supplement: Supplementary file 4 [file jcmm0018-1172-sd4.tif]
